# Supplementary material for: RHOA signaling defects result in impaired axon guidance in iPSC-derived neurons from patients with tuberous sclerosis complex
Source: Nat Commun. 2021 May 10;12:2589. doi: 10.1038/s41467-021-22770-4 (PMC8110792; doi:10.1038/s41467-021-22770-4)
Supplement: Supplementary file 1 — Supplemental Information [file 41467_2021_22770_MOESM1_ESM.pdf]

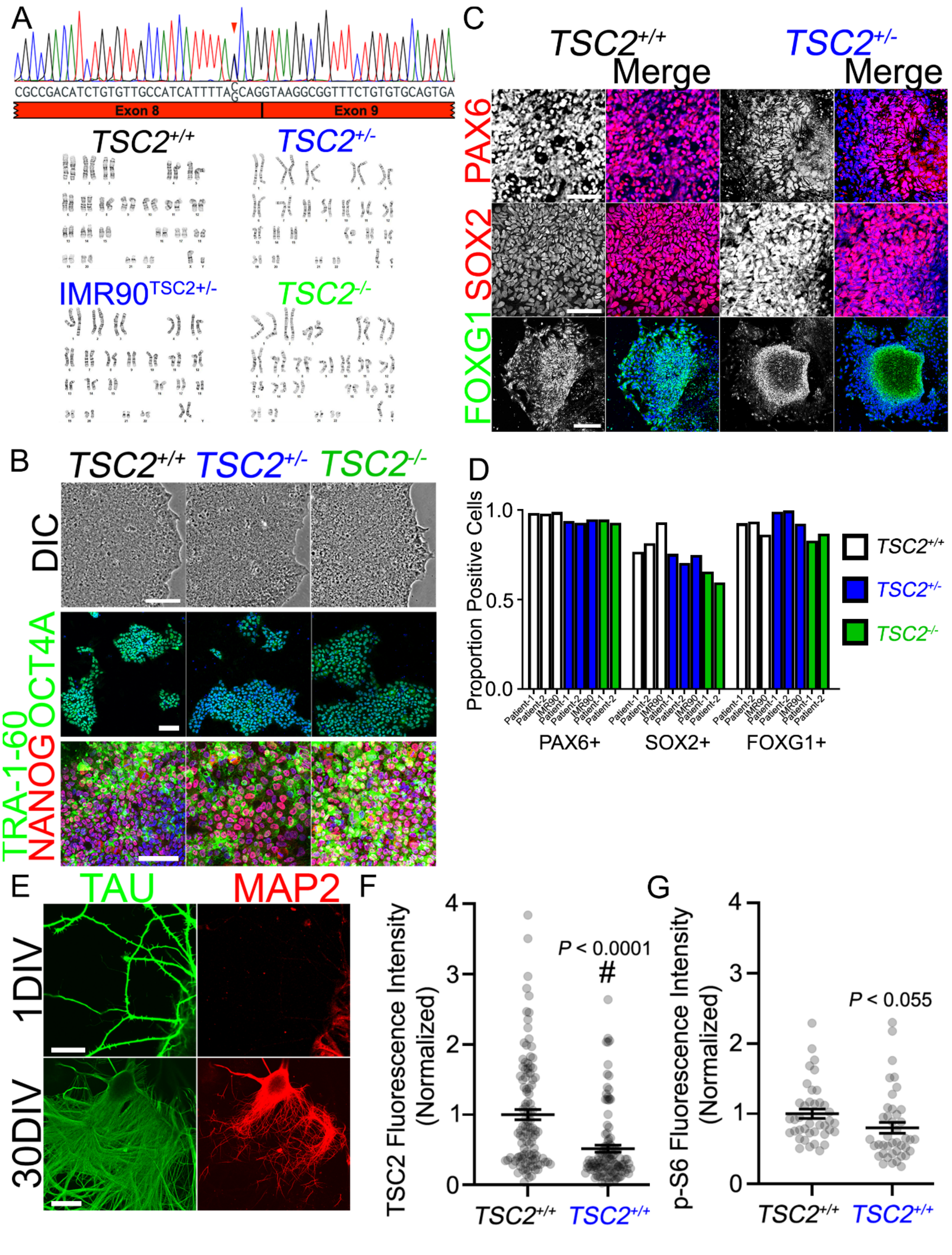

A
CGCCGACATCTGTGTTGCCATCATTTTA C/G CAGGTAAGGCGGTTTCTGTGTGCAGTGA
Exon 8
Exon 9
TSC2+/+
TSC2+/-
IMR90TSC2+/-
TSC2-/-
B
TSC2+/+
TSC2+/-
TSC2-/-
DIC
OCT4A
TRA-1-60
NANOG
C
TSC2+/+
Merge
TSC2+/-
Merge
PAX6
SOX2
FOXG1
D
Proportion Positive Cells
1.0
0.5
0.0
Patient-1
Patient-2
IMR90
PAX6+
SOX2+
FOXG1+
TSC2+/+
TSC2+/-
TSC2-/-
E
TAU
MAP2
1DIV
30DIV
F
TSC2 Fluorescence Intensity (Normalized)
P < 0.0001
#
TSC2+/+
TSC2+/+
G
p-S6 Fluorescence Intensity (Normalized)
P < 0.055
TSC2+/+
TSC2+/+

Supplemental Figure 1: *TSC2* mutant iPSCs and neurons express normal cell fate markers. A. Sanger sequencing of *TSC2*$^{+/-}$ iPSCs showed a nonsense mutation at bp 972 near the boundary between exon 8 and exon 9. Representative karyotypes shown from a clone of *TSC2*$^{+/+}$, patient-derived and IMR90 *TSC2*$^{+/-}$, and*TSC2*$^{-/-}$ genotypes, respectively. **B.** Reprogrammed iPSCs from patient lines as well as CRISPR-Cas9 corrected *TSC2*$^{+/+}$ and *TSC2*$^{-/-}$ cells were positive for pluripotency markers NANOG, OCT4, and TRA-1-60, and showed normal morphology and similar growth rates. **C-D**. Immunofluorescence for early neural (Sox2), progenitor (PAX6), and dorsal forebrain (FOXG1) fates showed appropriate neural differentiation in all cell lines. **E.** Co-staining for axonal marker TAU and dendritic marker MAP2 showed axonal specification at 1DIV in neurosphere preparations, and short dendritic processes in neurons migrating away from the neurosphere at 30DIV. **F-G.** Similar to patient-derived cells (Figure 1) in comparison to their isogenic controls, CRISPR-Cas9 generated IMR90$^{TSC2+/-}$ growth cones showed reduced TSC2 expression (P < 0.0001), as well as no significant difference in S6 activity relative to IMR90 controls (P < 0.055). # P < 0.0001. Two-tailed Student's t-test (F and G), represented as mean +/- s.e.m. Scale, 100 µm (B, C), 40 and 100 µm (E). Source data are provided as a Source Data file.

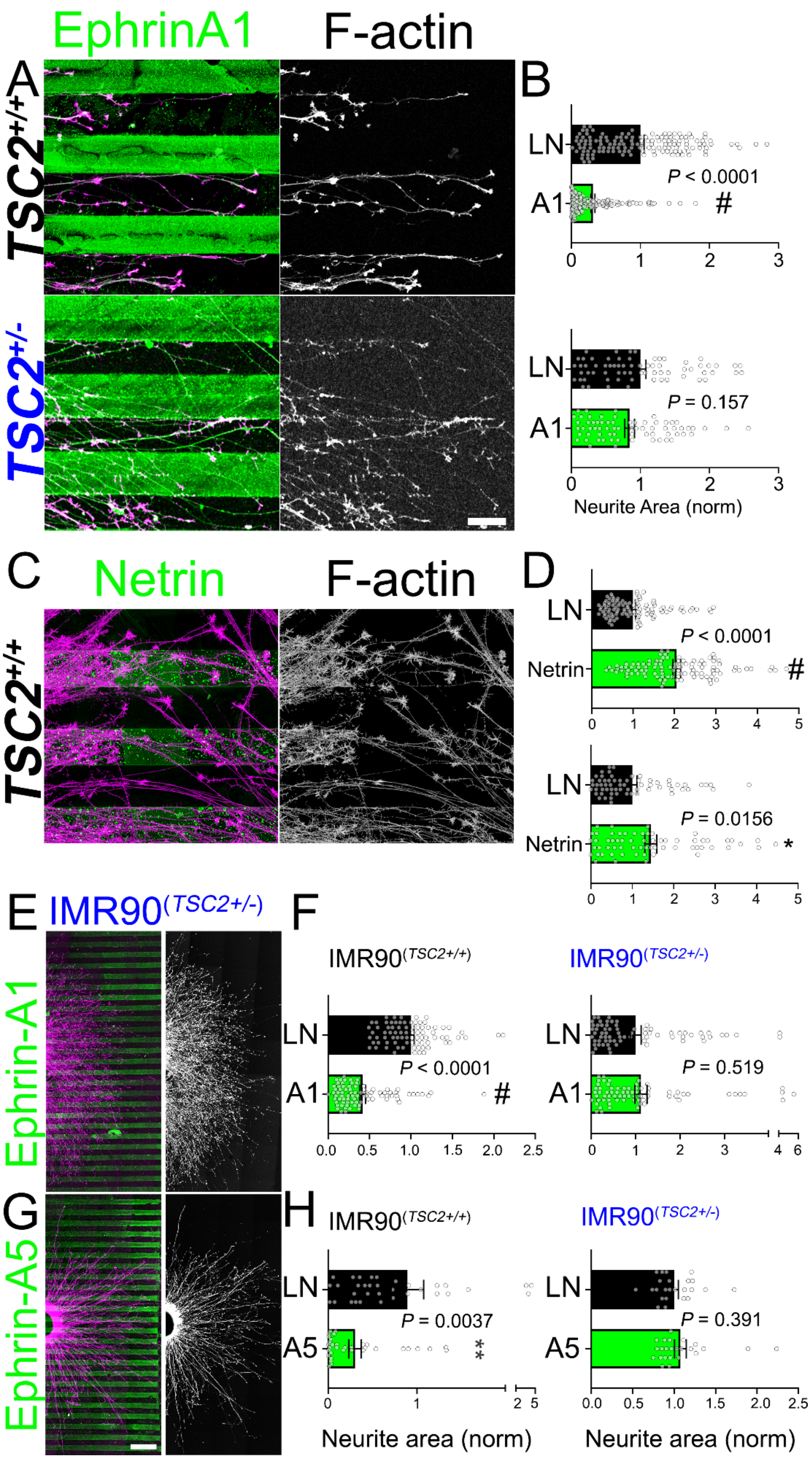

EphrinA1
F-actin
A
TSC2+/+
TSC2+/-
B
LN
A1
P < 0.0001
#
0
1
2
3
LN
A1
P = 0.157
Neurite Area (norm)
C
Netrin
F-actin
TSC2+/+
D
LN
Netrin
P < 0.0001
#
0
1
2
3
4
5
LN
Netrin
P = 0.0156
*
E
IMR90(TSC2+/-)
Ephrin-A1
F
IMR90(TSC2+/+)
IMR90(TSC2+/-)
LN
A1
P < 0.0001
#
0.0
0.5
1.0
1.5
2.0
2.5
P = 0.519
4
6
G
Ephrin-A5
H
LN
A5
P = 0.0037
**
P = 0.391
Neurite area (norm)
Neurite area (norm)

Supplemental Figure 2: Pathfinding by control hFB neurons along ephrin-A1 repulsive stripes is lost in *TSC2*$^{+/-}$ hFB neurons. **A, B.** High magnification images of LN/ephrin-A1 patterns (A) and analysis (B) of thresholded area occupied by neurites (F-actin channel) on each substratum normalized to the area of neurites growing on laminin only (see Methods). Note that *TSC2*$^{+/+}$ neurites robustly avoided Ephrin-A1 stripes ($P < 0.0001$), while *TSC2*$^{+/-}$ neurites failed to guide ($P = 0.157$). **C,D.** *TSC2*$^{+/+}$ ($P < 0.0001$) and *TSC2*$^{+/-}$ hFB neurites show a preference for Netrin-1 stripes ($P = 0.0156$). **E-H.** IMR90$^{TSC2+/-}$ neurites also cannot be guided by patterned ephrin-A1 ($P = 0.519$) (**E-F**) or ephrin-A5 ($P = 0.391$) (**G-H**) in comparison to IMR90 control neurospheres (ephrin-A1: $P < 0.0001$; ephrin-A5: $P = 0.0037$).* $P < 0.05$, ** $P < 0.01$, # $P < 0.0001$, Two-tailed Student's t-test, represented as mean +/- s.e.m. Scale, 100 μm. Source data are provided as a Source Data file.

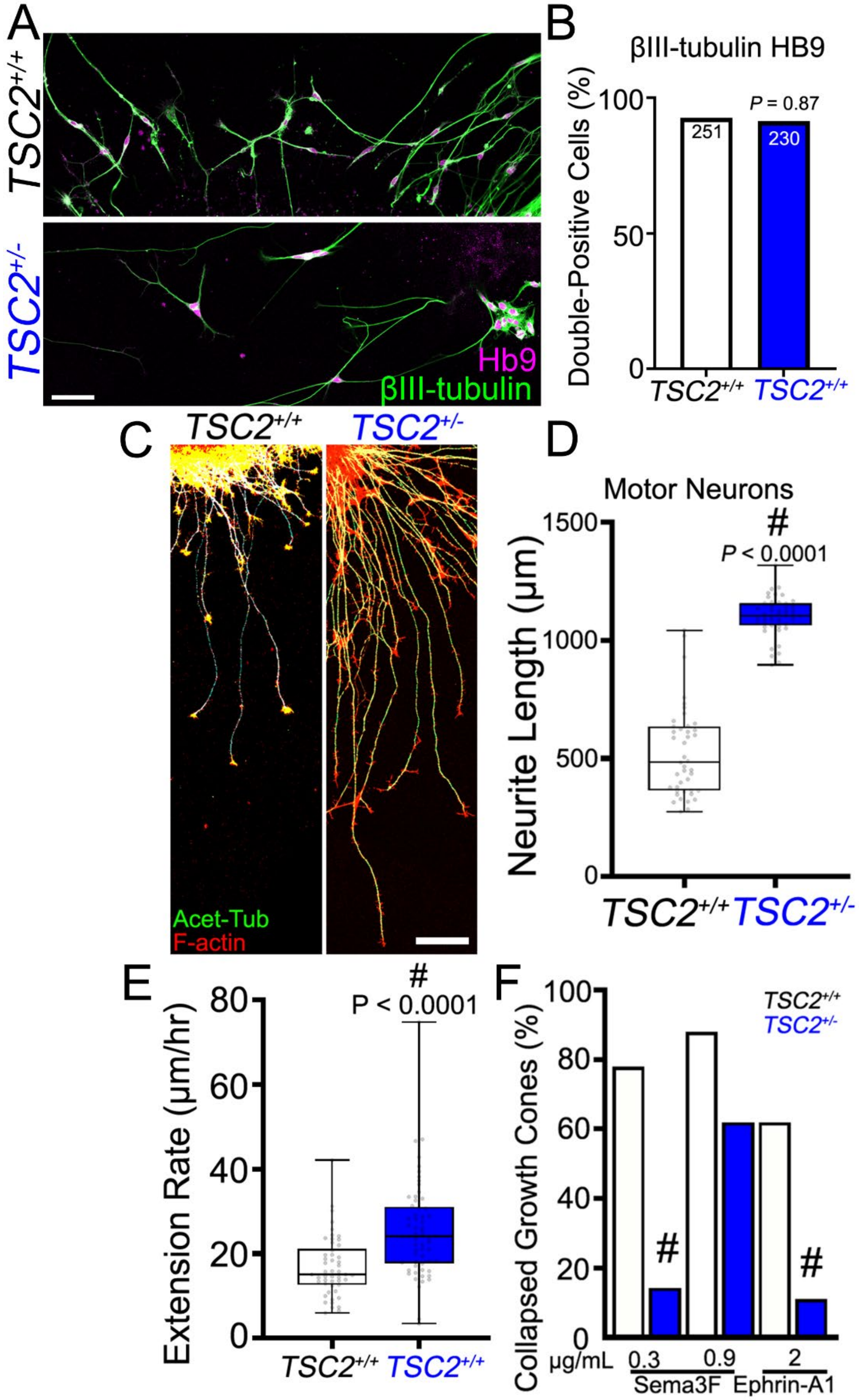
A
TSC2+/+
TSC2+/-
Hb9
βIII-tubulin
B
βIII-tubulin HB9
Double-Positive Cells (%)
100
50
0
P = 0.87
251
230
TSC2+/+
TSC2+/+
C
TSC2+/+
TSC2+/-
Acet-Tub
F-actin
D
Motor Neurons
Neurite Length (µm)
1500
1000
500
0
#
P < 0.0001
TSC2+/+
TSC2+/-
E
Extension Rate (µm/hr)
80
60
40
20
0
#
P < 0.0001
TSC2+/+
TSC2+/+
F
Collapsed Growth Cones (%)
100
80
60
40
20
0
TSC2+/+
TSC2+/-
#
#
µg/mL
0.3
0.9
2
Sema3F
Ephrin-A1

Supplemental Figure 3: *TSC2*$^{+/-}$ human motor neurons exhibit enhanced neurite extension compared to isogenic control neurons. A, B. Images and quantification of neurons co-labeled with neuron-specific βIII-tubulin and MN-specific HB9 antibodies. Quantification reflects % of βIII-tubulin-positive neurons co-labeled with HB9 (P = 0.87). **C.** hMN neurospheres of indicated genotype after one day *in vitro* were immunolabeled for acetylated-tubulin (green) and counter-stained for F-actin (red). Note longer axons extend from *TSC2*$^{+/-}$ neurospheres compared to isogenic control neurons. **D.** Average neurite lengths of 9 longest axons per neurosphere of each genotype (P < 0.0001). **E.** Average axon extension rates show that *TSC2*$^{+/-}$ hMN neurites grew markedly faster compared to isogenic control neurons (P < 0.0001). **F.** Analysis of percent growth cone collapse by *TSC2*$^{+/+}$ and *TSC2*$^{+/-}$ hMNs in response to Sema-3F (*TSC2*$^{+/+}$ vs. *TSC2*$^{+/-}$: high, P = 0.0024; low, P < 0.0001) and ephrin-A1 (*TSC2*$^{+/+}$ vs. *TSC2*$^{+/-}$, P < 0.0001). For MN experiments, ephrin-A1 was pre-clustered prior to application. Two-tailed Student's T-test, data represented as min, max, median, and IQR (D-E). Two-tailed Fisher's Exact Test (B,F). ** P < 0.05, # P < 0.0001. Scale, 100 µm. Source data are provided as a Source Data file.

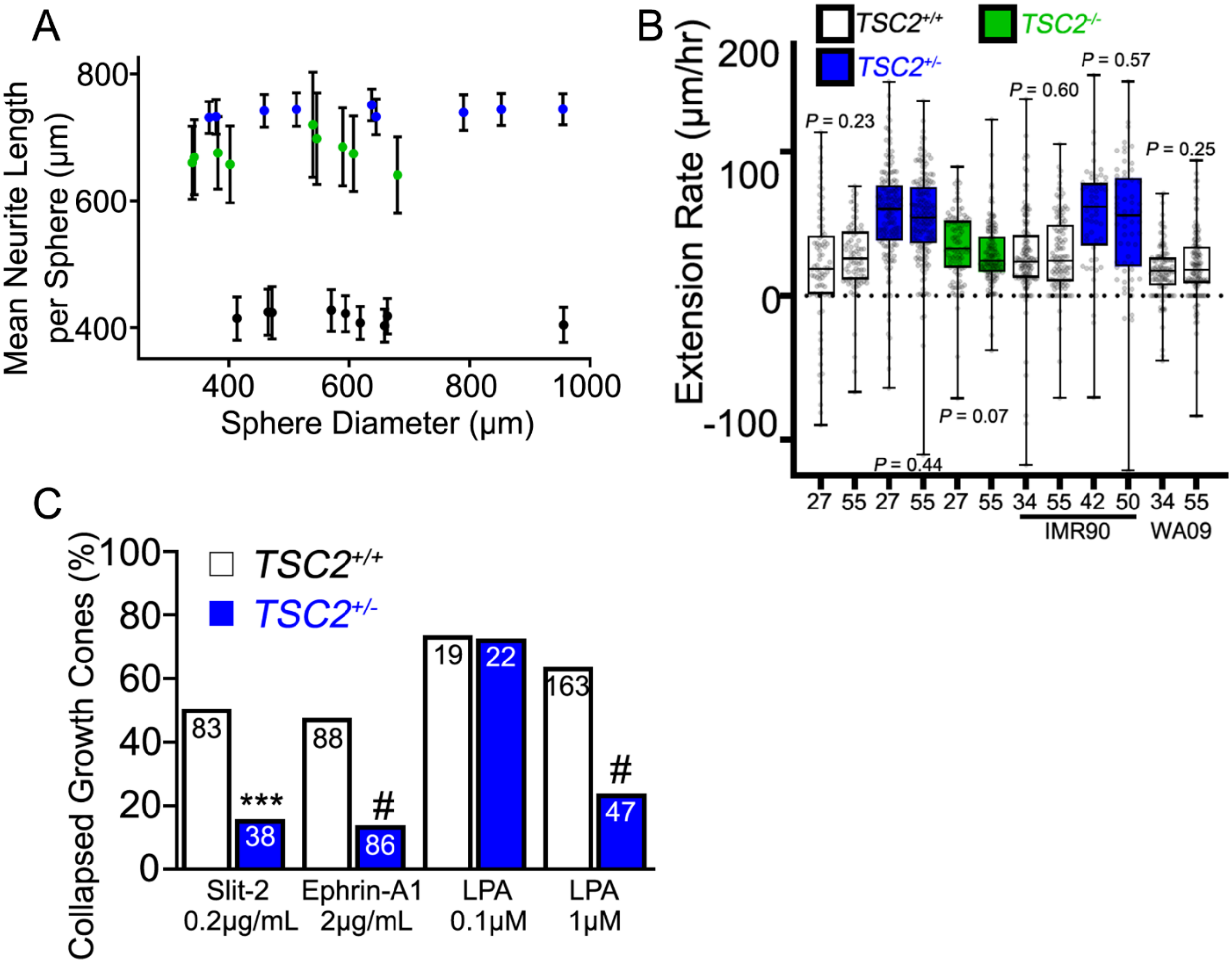

Supplemental Figure 4: Control cortical neurons differentiated from iPSC (IMR90) and ESC (WA09) lines are sensitive to inhibitory guidance cues. A. Mean neurite lengths from different sized neurospheres of each genotype were compared and no significant differences in outgrowth of spheres within each genotype were found between 200-1000 microns in diameter. **B.** Outgrowth rates of neurites of several cell lines were compared at early and late passage, and intra-line rates persist beyond passages utilized for experiments (*TSC2*$^{+/+}$, P = 0.23; *TSC2*$^{+/-}$, P = 0.44; *TSC2*$^{-/-}$, P = 0.07; IMR90, P = 0.60; IMR90$^{TSC2+/-}$, P = 0.57; WA09, P = 0.25). **C.** The percentage of collapsed hFB growth cones was compared between IMR90 control and IMR90$^{TSC2+/-}$

neurons. Measurements reflect collapse percentages after 15 min treatment with ephrin-A1 (2 μg/ml), Slit-2 (0.2 μg/ml), and LPA at low (0.1 μM) and high (1 μM) doses in neurosphere cultures. Changes in sensitivity are similar to that observed in patient-derived neurons in Figure 4F. Two-tailed Student's t-test, data represented as mean +/- s.e.m (A), and min, max, median, and IQR (B). Two-tailed Fisher's Exact Test (C). # P < 0.0001. Source data are provided as a Source Data file.

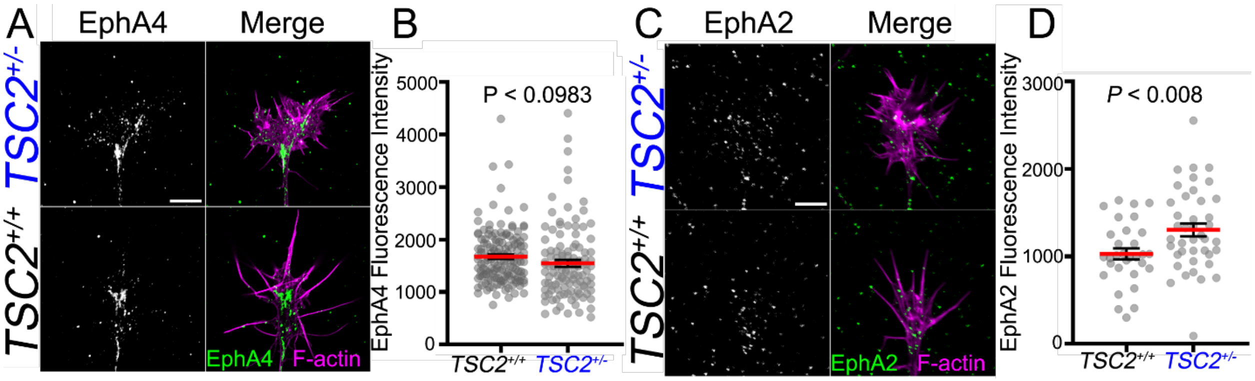

Supplemental Figure 5: *TSC2*$^{+/+}$ and *TSC2*$^{+/-}$ cortical neuronal growth cones express EphA4 and EphA2 receptors. A. hFB neurons were fixed and immunolabeled for EphA4 receptors (green in merge) and counterstained with phalloidin to label F-actin (magenta in merge). **B.** Fluorescence intensity measurements were made within growth cones and no significant difference was observed between *TSC2*$^{+/+}$ and *TSC2*$^{+/-}$ neurons (P = 0.0983). Growth cones from three independent experiments in *TSC2*$^{+/+}$ clone A and *TSC2*$^{+/-}$ clone A, respectively, were pooled for quantifications. **C.** hFB neurons were fixed and immunolabeled for EphA2 receptors (green in merge) and counterstained with phalloidin to label F-actin (magenta in merge). **D.** Fluorescence intensity measurements were made within growth cones and showed elevated EphA2 labeling in *TSC2*$^{+/-}$ neurons relative to *TSC2*$^{+/+}$ neurons (P = 0.008). Growth cones from two independent experiments in *TSC2*$^{+/+}$ clone A and *TSC2*$^{+/-}$ clone A, respectively,

were pooled for quantifications. Two-tailed Student's t-test, represented as mean +/- s.e.m. Scale, 5 µm. Source data are provided as a Source Data file.

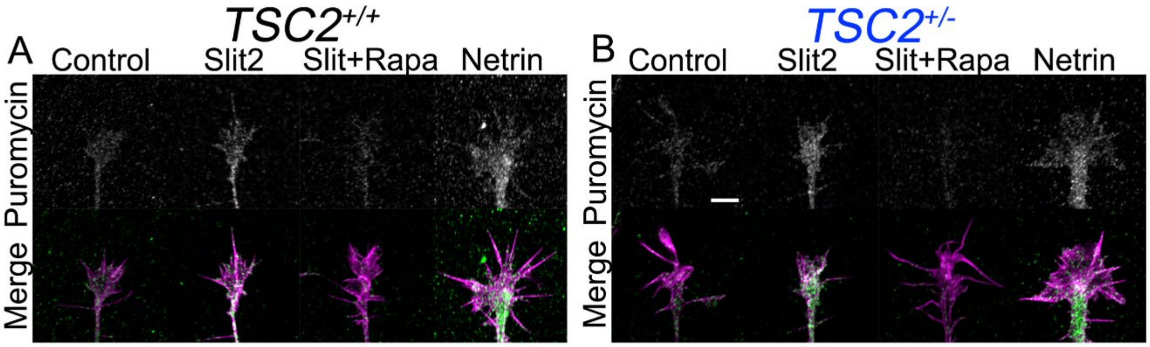

Supplemental Figure 6: Slit-2 and Netrin activate protein synthesis in *TSC2*$^{+/+}$ and *TSC2*$^{+/-}$ cortical growth cones. A, B. Puromycin labeled *TSC2*$^{+/+}$ (A) and *TSC2*$^{+/-}$ (B) hFB neuron growth cones (green in merges) counter-stained for F-actin (magenta in merges). Puromycin-labeled proteins were detected with anti-puromycin antibody (see methods). Note increased labeling in response to Slit-2 is blocked by pre-treatment with Rapamycin. Netrin treatment also increases puromycin incorporation. Quantification associated with these representative images are shown in Figure 5E and Supplementary Information. Scale, 5 µm. Source data are provided as a Source Data file.

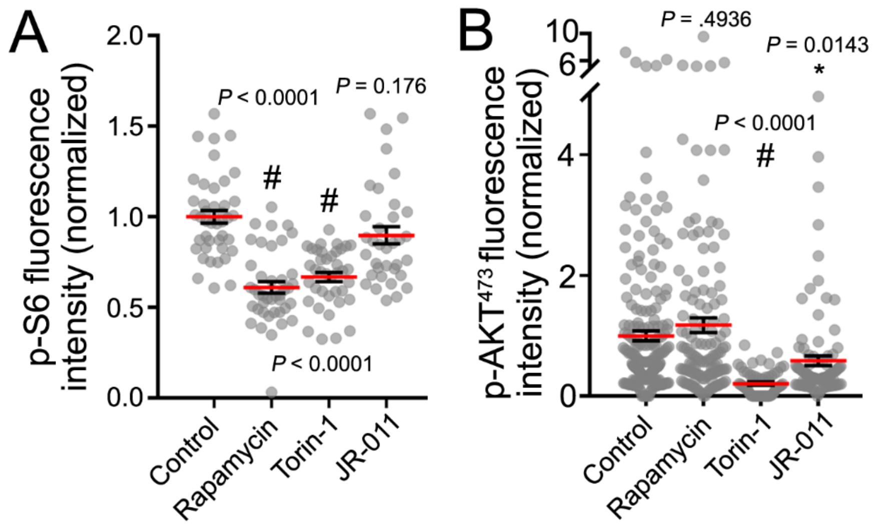

Supplemental Figure 7: Pharmacological inhibitors effectively block mTORC1 and mTORC2 function. hFB neurons were treated with mTOR inhibitors for 30 min then fixed and immunolabeled for p-S6 and p-AKT473, which are mTORC1- and mTORC2-specific targets, respectively. Fluorescence intensity measurements were made within growth cones and normalized to untreated control neurons (p-S6 Control vs. inhibitor: rapamycin, *P* < 0.0001; Torin-1, *P* < 0.0001; JR-AB2-011, *P* = 0.176. p-Akt Control vs. inhibitor: rapamycin, *P* < 0.4936; Torin-1, *P* < 0.0001; JR-AB2-011, *P* = 0.0143.). One-way ANOVA with Tukey's Multiple Comparison, represented as mean +/- s.e.m. * *P* < 0.05, # *P* < 0.0001. Source data are provided as a Source Data file.

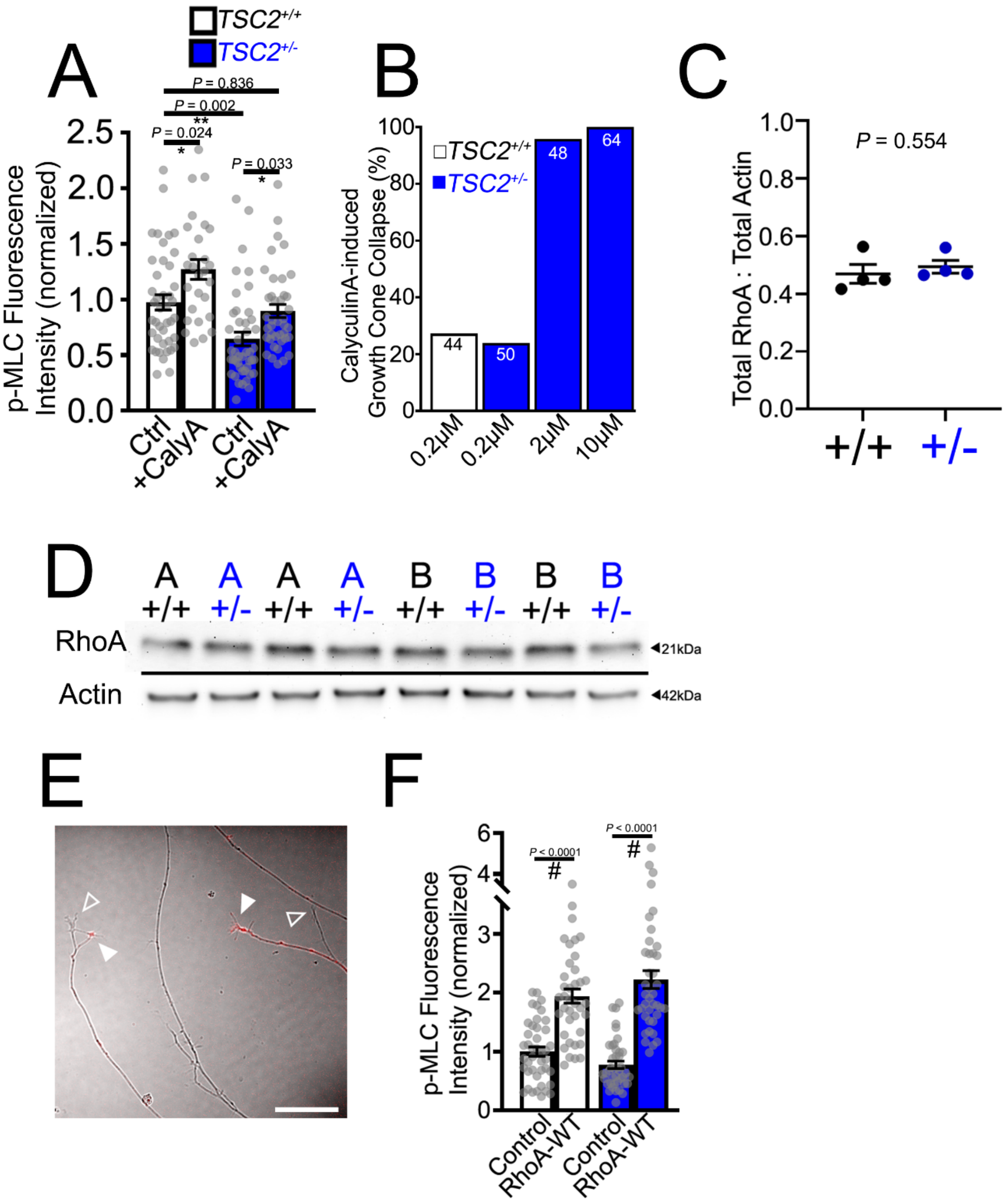

Supplemental Figure 8: Activation of p-MLC with calyculinA in growth cones. A. hFB neurons were treated with low dose of calyculinA (200pM) for 15 min then fixed and immunolabeled for p-MLC. Fluorescence intensity was measured within *TSC2*$^{+/+}$ and *TSC2*$^{+/-}$ growth cones and normalized to untreated control neurons. CalyA

activates p-MLC in both *TSC2*$^{+/+}$ and *TSC2*$^{+/-}$ growth cones (control vs. control, P = 0.002; *TSC2*$^{+/+}$ control vs. CalyculinA, P = 0.024; *TSC2*$^{+/-}$ control vs. CalyculinA, P = 0.033; *TSC2*$^{+/+}$ control vs. *TSC2*$^{+/-}$ CalyculinA, P = 0.836). Growth cones from two independent experiments in *TSC2*$^{+/+}$ clone A and *TSC2*$^{+/-}$ clone A, respectively, were pooled for quantifications. **B.** Calyculin was bath-applied to *TSC2*$^{+/+}$ and *TSC2*$^{+/-}$ growth cones at both collapsing and sub-collapsing doses. Collapse was assayed at 30min. Growth cones from two independent experiments in *TSC2*$^{+/+}$ clone A and *TSC2*$^{+/-}$ clone A, respectively, were pooled for quantifications. **C-D.** Western blot quantification and WB image of RHOA expression ratioed to total actin in *TSC2*$^{+/+}$ and *TSC2*$^{+/-}$ neurospheres (P = 0.554). Western blot shows data from two patient-derived clones and two independent differentiations. **E.** Representative image of lentiviral transduction in *TSC2*$^{+/-}$ neurites. Arrowheads show examples of RFP-positive neurites and empty arrowheads show RFP-negative neurites. Transduction rates with detectable RFP averaged 23% across 128 growth cones in 6 *TSC2*$^{+/+}$ neurospheres. **F.** Myosin activity within growth cones of *TSC2*$^{+/+}$ and *TSC2*$^{+/-}$ neurons transduced with RHOA-WT was increased relative to that of RFP-control transduced neurons in each genotype *(TSC2*$^{+/+}$ and *TSC2*$^{+/-}$: control vs RhoA-WT, P < 0.0001). Growth cones from two independent experiments in *TSC2*$^{+/+}$ clone A and *TSC2*$^{+/-}$ clone A, respectively, were pooled for quantifications. One-way ANOVA with Tukey's Multiple Comparison (A), or Two-tailed Student's t-test (C,F), represented as mean +/- s.e.m. # P < 0.0001. Source data are provided as a Source Data file.

Supplemental Table 1:

Figure	Panel	Genotype, Cell line, Neurosphere #, Growth Cone #
1	B	*TSC2+/+: 17 Neurospheres (NS) [6 Clone A (A), 11 Clone B (B)], 257 Growth cones (GCs), TSC2+/-: 12 NS (6A, 6B), 131 GCs, TSC2-/-: 5 NS (2 A, 3B), 27 GCs*
1	C	*TSC2+/+: 30 Neurospheres (NS), TSC2+/-: 30 NS, TSC2-/-: 30 NS*
1	E	*TSC2+/+: 7 NS (2 A, 5 B), 81 GCs, TSC2+/-: 7 NS (4A, 3B), 86 GCs, TSC2-/-: 5 NS (2 A, 3B), 74 GCs*
2	B	*TSC2+/+: 8 NS (4 A, 4 B), TSC2+/-: 10 NS (5 A, 5 B)*
2	D	*TSC2+/+ 12 NS (6 A, 6 B); TSC2+/- 14NS (4 A, 10 B)*
2	F	*TSC2+/+ 5NS*
2	H	*TSC2+/+ 4 NS (2 A, 2 B); TSC2+/- 5 NS (3 A, 2 B)*
3	B	*TSC2+/+: 23 NS (16 A, 7 B), 460 GCs; TSC2+/-: 13 NS (3 A, 10 B), 260 GCs; TSC2-/-: 9 NS (5 A, 4 B), 180 GCs*
3	D	*TSC2+/+: 5 NS (2 A, 3 B), TSC2+/-: 10 NS (5 A, 5 B), TSC2-/-: 9 NS (5 A, 4 B)*
4	B	*TSC2+/+ 3 NS (2 A, 1 B), 15 GCs; TSC2+/- 2 NS (1 A, 1 B), 22 GCs*
4	C	*TSC2+/+ 4 NS (2 A, 2 B), 24 GCs; TSC2+/- 2 NS (1 A, 1 B), 10 GCs*
4	F	Slit-2: TSC2+/+ 7 NS (4 A, 3 B), 89 GCs; TSC2+/- 5 NS (2 A, 3 B), 50 GCs; ephrin low: TSC2+/+ 22 NS (18 A, 4 B), 313 GCs; TSC2+/- 12 NS (7 A, 5 B) 78 GCs; ephrin high TSC2+/+ 26 NS (20 A, 6 B), 352 GCs; TSC2+/- 7 NS (2 A, 5 B), 86 GCs; LPA low TSC2+/+ 14 NS (6 A, 8 B), 199 GCs; TSC2+/- 12 NS (6 A, 6 B), 201 GCs; LPA high TSC2+/+ 4 NS (2 A, 2 B) 28 GCs; TSC2+/- 4 NS (2 A, 2 B), 22 GCs; control TSC2+/+ 9 NS (2 A, 7 B), 114 GCs; TSC2+/- 6 NS (2 A, 4 B), 89 GCs
5	B	Control: TSC2+/+ 9 NS (5 A, 4 B), 98 GCs; TSC2+/- 12 NS (7 A, 5 B), 110 GCs; TSC2-/- 8 NS (4 A, 4 B), 81 GCs; Rapa: TSC2+/+ 3 NS (2 A, 1 B), 41 GCs; TSC2+/- 6 NS (3 A, 3 B), 42 GCs; TSC2-/- 8 NS (4 A, 4 B), 64 GCs; Aniso: TSC2+/+ 4 NS (4 A), 45 GCs
5	D	Control: TSC2+/+ 9 NS (5 A, 4 B), 79 GCs; TSC2+/- 6 NS (3 A, 3 B), 95 GCs; TSC2-/- 4 NS (2 A, 2 B), 42 GCs; ephrin: TSC2+/+ 6 NS (2 A, 4 B), 67 GCs; TSC2+/- 10 NS (8 A, 2 B), 120 GCs; TSC2-/- 5 NS (2 A, 3 B), 34 GCs
5	E	Control: TSC2+/+ 9 NS (4 A, 5 B), 105 GCs; TSC2+/- 10 NS (2 A, 8 B), 136 GCs; Slit-2: TSC2+/+ 7 NS (5 A, 2 B), 119 GCs; TSC2+/- 12 NS (6 A, 6 B) 110 GCs; Slit-2 + Rapa: TSC2+/+ 7 NS (3 A, 4 B), 43 GCs; TSC2+/- 7 NS (3 A, 4 B), 84 GCs; Netrin: TSC2+/+ 4 NS (2 A, 2 B), 45 GCs; TSC2+/- 8 NS (6 A, 2 B), 100 GCs
6	A	Control: TSC2+/+ 10 NS (4 A, 6 B), 110 GCs; TSC2+/- 10 NS (6 A, 4 B), 108 GCs; Torin1: TSC2+/+ 7 NS (4 A, 3 B), 55 GCs; TSC2+/- 12 NS (6 A, 6 B) 94 GCs; Rapa: TSC2+/+ 4 NS (2 A, 2 B), 44 GCs; TSC2+/- 5 NS (2 A, 3 B), 40 GCs; JR-011: TSC2+/- 4 NS (2 A, 2 B), 42 GCs
6	B	Control: TSC2+/+ 8 NS (6 A, 2 B), 80 GCs; TSC2+/- 5 NS (2 A, 3 B), 58 GCs; Torin1: TSC2+/+ 7 NS (5 A, 2 B), 93 GCs; TSC2+/- 6 NS (2 A, 4 B) 47 GCs; Rapa: TSC2+/+ 4 NS (2 A, 2 B), 82 GCs; TSC2+/- 6 NS (4 A, 2 B), 37 GCs; Rapa 4DIV: TSC2+/- 5 NS (2 A, 3 B), 42 GCs; Rapa 21DIV: TSC2+/- 4 NS (2 A, 2 B), 45 GCs
6	C	Control: TSC2+/+ 6 NS (3 A, 3 B), 84 GCs; TSC2+/- 18 NS (15 A, 3 B), 198 GCs; Torin1: TSC2+/+ 9 NS (7 A, 2 B), 80 GCs; TSC2+/- 5 NS (3 A, 2 B) 69 GCs; Rapa: TSC2+/+ 7 NS (5 A, 2 B), 91 GCs; TSC2+/- 6 NS (4 A, 2 B), 109 GCs; JR-011: TSC2+/- 5 NS (3 A, 2 B), 64 GCs
6	D	Control: TSC2+/+ 17 NS (14 A, 3 B), 129 GCs; TSC2+/- 9 NS (2 A, 7 B), 99 GCs; Torin1: TSC2+/+ 8 NS (3 A, 5 B), 122 GCs; TSC2+/- 11 NS (5 A, 6 B) 72 GCs; Rapa: TSC2+/+ 9 NS (3 A, 6 B), 127 GCs; TSC2+/- 4 NS (2 A, 2 B), 73 GCs; Rapa 4DIV: TSC2+/- 6 NS (2 A, 4 B), 90 GCs; Rapa 21DIV: TSC2+/- 5 NS (3 A, 2 B), 70 GCs
6	F	*TSC2+/+ 11 NS (7 A, 4 B); TSC2+/- 8 NS (3 A, 5 B)*
6	H	*TSC2+/+ 10 NS (3 A, 7 B); TSC2+/- 5 NS (2 A, 3 B)*
7	B	*TSC2+/+ 10 NS (A), 18 GCs; TSC2+/- 9 NS (A), 13 GCs*
7	C	*Control: TSC2+/+ 6 groups of 30 NS (3A, 3B); TSC2+/- 6 groups of 30 NS (3A, 3B), ephrin: TSC2+/+ 4 groups of 30 NS, TSC2+/- 4 groups of 30 NS (2A, 2B)*
7	F	Control: TSC2+/+ 9 NS (3A, 6B), 64 GCs; TSC2+/- 6 NS (2A, 4B), 69 GCs; Ephrin: TSC2+/+ 9 NS (3A, 6B), 84 GCs; TSC2+/- 10 NS (5A, 5B), 84 GCs

8	B	Control: TSC2+/+ 4 NS (2 A, 2 B), 61 GCs; TSC2+/- 5 NS (3 A, 2 B), 51 GCs; Y27632: TSC2+/+ 6 NS (4 A, 2 B), 58 GCs; TSC2+/- 4 NS (2 A, 2 B) 30 GCs; Blebb: TSC2+/+ 4 NS (2 A, 2 B), 25 GCs; TSC2+/- 4 NS (2 A, 2 B), 30 GCs; CalyA: TSC2+/+ 4 NS (2 A, 2 B), 39 GCs; TSC2+/- 5 NS (3 A, 2 B), 39 GCs; Control-lenti: TSC2+/- 7 NS (4 A, 3 B), 54 GCs; RHOA-lenti: TSC2+/- 7 NS (3 A, 4 B), 34 GCs
8	C	Control: TSC2+/+ 4 NS (2 A, 2 B), 62 GCs; TSC2+/- 4 NS (2 A, 2 B), 38 GCs; Y27632: TSC2+/+ 4 NS (2 A, 2 B), 45 GCs; TSC2+/- 4 NS (2 A, 2 B) 53 GCs; C3 Transferase: TSC2+/+ 6 NS (2 A, 4 B), 50 GCs; TSC2+/- 5 NS (2 A, 3 B) 49 GCs; Blebb: TSC2+/+ 5 NS (2 A, 3 B), 48 GCs; TSC2+/- 6 NS (3 A, 3 B), 74 GCs; CalyA: TSC2+/+ 7 NS (5 A, 2 B), 62 GC TSC2+/- 6 NS (3 A, 3 B), 62 GCs; Control-lenti: TSC2+/- 5 NS (3 A, 2 B), 58 GCs; RHOA-lenti: TSC2+/- 5 NS (3 A, 2 B), 59 GCs
8	E	Y-27632: TSC2+/+ 10 NS (4A, 6B); TSC2+/- 4 NS (2A, 2B)
S1	D	*TSC2+/+ patient clone 1: (PAX6 704 cells, SOX2 666 cells, FOXG1 734 cells); TSC2+/+ patient clone 2: (PAX6 328 cells, SOX2 868 cells, FOXG1 298 cells); TSC2+/+ IMR90: (PAX6 548 cells, SOX2 593 cells, FOXG1 226 cells); TSC2+/- patient clone 1: (PAX6 262 cells, SOX2 1286 cells, FOXG1 311 cells); TSC2+/- patient clone 2: (PAX6 397 cells, SOX2 470 cells, FOXG1 348 cells); TSC2+/- IMR90: (PAX6 384 cells, SOX2 434 cells, FOXG1 139 cells); TSC2-/- patient clone 1: (PAX6 398 cells, SOX2 381 cells, FOXG1 7233 cells); TSC2-/- patient clone 2: (PAX6 185 cells, SOX2 274 cells, FOXG1 350 cells);*
S1	F	*IMR90-TSC2+/+: 8 NS, 109 GCs, IMR90-TSC2+/-: 9 NS, 109 GCs*
S1	G	*IMR90-TSC2+/+: 3 NS, 41 GCs, IMR90-TSC2+/-: 5 NS, 39 GCs*
S2	B	*TSC2+/+ 13 NS (8 A, 5 B); TSC2+/- 12 NS (5 A, 7 B)*
S2	D	*TSC2+/+ 6 NS (3 A, 3 B); TSC2+/- 4 NS (2 A, 2 B)*
S2	F	IMR90-TSC2+/+ 6 NS; TSC2+/- 7 NS
S2	H	IMR90-TSC2+/+ 3 NS; TSC2+/- 5 NS
S3	B	*TSC2+/+ 251 neurons; TSC2+/- 230 neurons*
S3	D	*TSC2+/+ 4 NS, 43 GCs; TSC2+/- 5NS, 47 GCs*
S3	E	*TSC2+/+ 2 NS, 50 GCs; TSC2+/- 2 NS, 61 GCs*
S3	F	Sema-3F low: TSC2+/+ 4 NS, 50 GCs; TSC2+/- 4 NS, 56 GCs; Sema-3F high: TSC2+/+ 5 NS, 50 GCs; TSC2+/- 5 NS, 63 GCs; ephrin TSC2+/+ 4 NS, 63 GCs; TSC2+/- 5 NS, 54 GCs
S4	A	Patient lines, all genotypes: 9 NS, 180 GCs
S4	B	IMR90-TSC2+/+ p34: 6 NS, 130 GCs; p55: 8 NS, 112 GCs; TSC2+/- p43: 3 NS, 57 GCs; p50 4 NS, 60 GCs; H9 p34: 10 NS, 109 GCs, p55: 8 NS, 118 GCs; Patient lines: TSC2+/+ p27: 5 NS, 78 GCs, p55: 6NS, 77 GCs; TSC2+/- p27: 9 NS, 153 GCs, p55: 10NS, 167 GCs; TSC2-/- p27: 5 NS, 93 GCs, p55: 11 NS, 129 GCs
S4	C	Slit-2: IMR90-TSC2+/+ 7 NS, 83 GCs; TSC2+/- 4 NS, 38 GCs; ephrin: TSC2+/+ 6 NS, 88 GCs; TSC2+/- 12 NS, 86 GCs; LPA high TSC2+/+ 2 NS, 20 GCs; TSC2+/- 2 NS, 23 GCs; LPA low TSC2+/+ 15 NS, 163 GCs; TSC2+/- 5 NS, 46 GCs
S5	B	*TSC2+/+ 7 NS, 144 GCs, TSC2+/- 3 NS, 111 GCs*
S5	D	*TSC2+/+, 2NS, 31 GCs, TSC2+/-, 2 NS, 41 GCs*
S7	A	p-S6: Control 4 NS, 42 GCs; Rapa 4 NS, 41 GCs; Torin-1 4 NS, 39 GCs, JR-011 4NS, 35 GCs
S7	B	p-Akt: Control 15 NS, 195 GCs; Rapa 14 NS, 132 GCs; Torin-1 4 NS, 60 GCs, JR-011 7 NS, 99 GCs
S8	A	*TSC2+/+ Control: 2NS, 42 GCs; CalyA: 2 NS, 27 GCs; TSC2+/- Control: 2 NS, 44 GCs, CalyA 3 NS, 40 GCs*
S8	B	*TSC2+/+ 200pM: 3 NS, 44 GCs; TSC2+/- 200pM: 3 NS, 50 GCs, 2uM: 4 NS, 48 GCs, 10 uM: 4 NS, 64 GCs*
S8	C	Each group: 30 NS
S8	E	*TSC2+/+*, 6 NS, 128 GCs
S8	F	*TSC2+/+ Control-lenti: 3NS, 43 GCs; RHOA-WT: 3 NS, 38 GCs; TSC2+/- Control-lenti: 2 NS, 42 GCs, RHOA-WT 2 NS, 40 GCs*

Summary of experimental replicates by genotype, cell line, and clone. Neurospheres (NS), clone A (A), clone (B), and genotype are indicated. Minimum two differentiations per experiment, 1-5 neurospheres per experimental group per differentiation.

Supplemental Table 2: Primers

**sgRNA cloning**	
TSC2 induction sgRNA 1 F	CACCGCACACAGAAACCGCCTTACC
TSC2 induction sgRNA 1 R	AAACGGTAAGGCGGTTTCTGTGTGC
TSC2-correction (-) F	CACCGCGCCTTACCTGCTAAAATGA
TSC2-correction (-) R	AAACTCATTTTAGCAGGTAAGGCGC
**ssODN**	
TSC2 correction ssODN	ATGGGTGGGCAGGCGTGAGGGGAGCTCTCCCGTTCCTGCCAGCTCACTGCACACAGAA ACCGCCTTACCTGGTAAAATGATGGCAACACAGATGTCGGCGAGTTCCTGAGAGAATAG AGCCGGTGGGCTCCCCAGAGAGCCA
TSC2 induction ssODN	ATGGGTGGGCAGGCGTGAGGGGAGCTCTCCCGTTCCTGCCAGCTCACTGCACACAGAA ACCGCCTTACCTGCTAAAATGATGGCAACACAGATGTCGGCGAGTTCCTGAGAGAATAG AGCCGGTGGGCTCCCCAGAGAGCCA
**Genotyping primers**	
TSC2 genotyping 2F	TGTGGCTTTCCTTCTTGGCT
TSC2 genotyping 2R	CTACAAAGGGCCCCAAACCT
**Off-target analysis primers**	
TSC2 Induction OT 1F	CCCCAGTGTCTAGCATAGCG
TSC2 Induction OT 1R	GTGAAGAGCTTTCTCTTCCATTTCA
TSC2 Induction OT 2F	CTGAGCCAACCTAAGGTCCC
TSC2 Induction OT 2R	AACCATGCAAGTGAGGGAGG
TSC2 Induction OT 3F	TGTCTCTGAAAAATGAAGACGTGA
TSC2 Induction OT 3R	TGGGGCAGTTTGGGACAATG
TSC2 Induction OT 4F	TTGGTGTGTTTGGGGCTTCT
TSC2 Induction OT 4R	ACCAAAGCCAGATAACCGGG
TSC2 Induction OT 5F	ACCTCATCAGCGTGCATACC
TSC2 Induction OT 5R	GTGAGTGCAGAAGCTGGAGT
TSC2 correction OT 1F	GACCCAGCAGTGTATTCCCC
TSC2 correction OT 1R	AGCTGTGGACTGTCTCTGGA
TSC2 correction OT 2F	TCTGTTCTCCCACATGCCAC
TSC2 correction OT 2R	TGCTAGACCCAGAGACCCAG
TSC2 correction OT 3F	GATGGCGAGACTCACTCCTG
TSC2 correction OT 3R	CGTCTCACCTCCCTCCCTTA
TSC2 correction OT 4F	TCCCGCAATGCCCATATAGC
TSC2 correction OT 4R	TGATGTCTGTTGGAACCCCAG
TSC2 correction OT 5F	CTGCAGCCTCCTTGGAGAAA
TSC2 correction OT 5R	CACCCTGGCAAGCAATGTTC

Supplemental Table 3: Antibodies

Vendor	Antibody	Item ID #	Assay & Dilution	Verification
MP Biomedicals	Actin	8691001	WB (1:5000)	Actin: Verified in our lab via WB band at appropriate size.
Sigma	beta-III tubulin	T8660-100UL	IF (1:500)	beta-III tubulin: by peer review, >360 citations
Cell Signaling	EphA2(D4A2)	6997T	IF (1:250)	EphA2: by manufacturer, via EphA2 expressing and non-expressing cell lines, respectively.
Jackson Immunoresearch	Fc	109-005-098	IF (1:250)	Anti-Fc: Verified in our lab via staining of Fc protein carpets.
Abcam	FOXG1	ab18259	IF (1:200)	FoxG1: by manufacturer, via FoxG1 overexpression in embryonic cortex.
DSHB	Hb9 Mnx1	81.5C10-c	IF (1:40)	Hb9: by depositor, Tanabe et al., Cell 1998
Sigma	MAP2	M4403	IF (1:500)	MAP2: by manufacturer immunofluorescent labeling specific to dendritic processes
R&D Systems	mEphA4	AF641	IF (1:250)	EphA4: by manufacturer, via specific staining of embryonic rat rib cartilage primordium.
Cell Signaling	Nanog (D73G4)	4903T	IF (1:200)	Nanog: by manufacturer, via immunofluorescent staining of pluripotent and differentiated cells
Cell Signaling	Oct-4A (C20A3)	2840T	IF (1:400)	Oct4a: by manufacturer, via immunofluorescent staining of pluripotent and differentiated cells
DSHB	PAX6	PAX6	IF (1:40)	Pax6: by depositor, Kawakami et al., Mech Dev 1997.
Cell Signaling	phospho-Akt (S473)	4060S	IF (1:400)	p-Akt: by manufacturer, via PI3K inhibition and insulin treatments, respectively.
ThermoFisher	phospho-MYL9 (Thr18, Ser19)	PA5-17727	IF (1:250)	p-MYL9: by manufacturer, via co-labeling w/ actin in HeLa cells.
Cell Signaling	phospho-S6 ribosomal protein (S235/236)	48585	WB (1:1000); IF (1:100)	p-S6: by manufacturer, via rapamycin and serum treatment, respectively.
Cell Signaling	phospho-S6 ribosomal protein (S235/236)	62016S	IF (1:400)	p-S6: by manufacturer, via rapamycin and serum treatment, respectively.
DSHB	puromycin	PMY-2A4-s	IF (1:40)	Puromycin: Verified in our lab via protein synthesis inhibition and agonist experiments.
Cell Signaling	RhoA	2117	WB (1:1000)	RhoA: by manufacturer, via cell line overexpression and WB assay
Cell Signaling	S6 ribosomal protein	2317S	WB (1:1000); IF (1:50)	S6: by manufacturer, via WB band at appropriate size
R&D Systems	SOX2	AF2018	IF (1:250)	Sox2: by manufacturer, via specific staining in multiple neural-differentiated stem cell lines.
Dako	Tau	A0024	IF (1:500)	Tau: by peer review, >160 citations.
Invitrogen	Tra-1-60	ma1-023	IF (1:75)	Tra-1-60: by manufacturer, via immunofluorescent staining of pluripotent and differentiated cells
Cell Signaling	Tuberin/TSC2	3635	WB (1:1000); IF (1:400)	TSC2: Verified in our lab via staining patterns in WT and TSC2(-/-) cell lines
Sigma	tubulin, acetylated	T6793-2ML	IF (1:500)	Acetylated-tubulin: by peer review, >700 citations.
Jackson Immunoresearch	HRP – anti-Rabbit	211-032-171	WB (1:10000)	HRP: by peer review, > 200 citations
