# Supplementary material for: RHOA signaling defects result in impaired axon guidance in iPSC-derived neurons from patients with tuberous sclerosis complex
Source: Nat Commun. 2021 May 10;12:2589. doi: 10.1038/s41467-021-22770-4 (PMC8110792; doi:10.1038/s41467-021-22770-4)
Supplement: Supplementary file 3 — Reporting Summary [file 41467_2021_22770_MOESM3_ESM.pdf]

nature research

Corresponding author(s): Timothy Gomez

Last updated by author(s): 03/25/2021

Reporting Summary

Nature Research wishes to improve the reproducibility of the work that we publish. This form provides structure for consistency and transparency in reporting. For further information on Nature Research policies, see Authors & Referees and the Editorial Policy Checklist .

Statistics

For all statistical analyses, confirm that the following items are present in the figure legend, table legend, main text, or Methods section.

n/a	Confirmed	
[ ]	[x]	The exact sample size (*n*) for each experimental group/condition, given as a discrete number and unit of measurement
[ ]	[x]	A statement on whether measurements were taken from distinct samples or whether the same sample was measured repeatedly
[ ]	[x]	The statistical test(s) used AND whether they are one- or two-sided<br>*Only common tests should be described solely by name; describe more complex techniques in the Methods section.*
[ ]	[x]	A description of all covariates tested
[ ]	[x]	A description of any assumptions or corrections, such as tests of normality and adjustment for multiple comparisons
[ ]	[x]	A full description of the statistical parameters including central tendency (e.g. means) or other basic estimates (e.g. regression coefficient) AND variation (e.g. standard deviation) or associated estimates of uncertainty (e.g. confidence intervals)
[ ]	[x]	For null hypothesis testing, the test statistic (e.g. *F*, *t*, *r*) with confidence intervals, effect sizes, degrees of freedom and *P* value noted<br>*Give P values as exact values whenever suitable.*
[x]	[ ]	For Bayesian analysis, information on the choice of priors and Markov chain Monte Carlo settings
[x]	[ ]	For hierarchical and complex designs, identification of the appropriate level for tests and full reporting of outcomes
[x]	[ ]	Estimates of effect sizes (e.g. Cohen's *d*, Pearson's *r*), indicating how they were calculated

Our web collection on statistics for biologists contains articles on many of the points above.

Software and code

Policy information about availability of computer code

Data collection	Images were collected via Zen (v3.1) or Metamorph (v7.7.2.0)
Data analysis	FIJI/ImageJ v1.51 was used for all data analysis.

For manuscripts utilizing custom algorithms or software that are central to the research but not yet described in published literature, software must be made available to editors/reviewers. We strongly encourage code deposition in a community repository (e.g. GitHub). See the Nature Research guidelines for submitting code & software for further information.

Data

Policy information about availability of data

All manuscripts must include a data availability statement. This statement should provide the following information, where applicable:

- Accession codes, unique identifiers, or web links for publicly available datasets
- A list of figures that have associated raw data
- A description of any restrictions on data availability

The data supporting the findings of this study are available within the article, its Supplementary Figures, and source data file.

Field-specific reporting

Please select the one below that is the best fit for your research. If you are not sure, read the appropriate sections before making your selection.

[x] Life sciences [ ] Behavioural & social sciences [ ] Ecological, evolutionary & environmental sciences

For a reference copy of the document with all sections, see nature.com/documents/nr-reporting-summary-flat.pdf

Life sciences study design

All studies must disclose on these points even when the disclosure is negative.

Sample size	Typically for live extension/collapse and fixed immunofluorescence experiments, we imaged a minimum of 10 growth cones from four or more different neurospheres (>40 growth cones), pooling data together from two separate differentiations per genotype. For stripe assays, experiments were quantified from a minimum of 10 ROIs parallel to a minimum of five neurospheres on a minimum of three separate stripe substrates from two separate differentiations. A 95% confidence interval was selected as significant. No statistical methods were used to predetermine sample size.
Data exclusions	No data were excluded.
Replication	Experiments were replicated using a minimum of four neurospheres from two independent differentiations. Similar results were observed upon replication. Data from individual clones were pooled for genotype comparisons except where noted. The experiments in this manuscript were matched to within ten passages. Key experiments were repeated at early (p18-33, depending on the cell line) and late (p50-60) passages, confirming phenotype consistency across passage number. Individual clone information is included within the Source Data file.
Randomization	Randomization was not relevant to this study, as groups differed only by genotype and no discrimination was made between them - each was tested independently in all experiments.
Blinding	Investigators were blinded to experimental groups during image acquisition in preliminary experiments, as well as to experimental groups during image analysis.

Reporting for specific materials, systems and methods

We require information from authors about some types of materials, experimental systems and methods used in many studies. Here, indicate whether each material, system or method listed is relevant to your study. If you are not sure if a list item applies to your research, read the appropriate section before selecting a response.

Materials & experimental systems

n/a	Involved in the study	
☐	☒	Antibodies
☐	☒	Eukaryotic cell lines
☒	☐	Palaeontology
☒	☐	Animals and other organisms
☐	☒	Human research participants
☒	☐	Clinical data

Methods

n/a	Involved in the study	
☒	☐	ChIP-seq
☒	☐	Flow cytometry
☒	☐	MRI-based neuroimaging

Antibodies

Antibodies used

MP Biomedicals Actin Mouse IgG1 monoclonal (C4) 8691001
Sigma beta-III tubulin Mouse IgG2b monoclonal (SDL.3010) T8660-100UL
Cell Signaling EphA2 Rabbit IgG monoclonal (D4A2) 6997T
Jackson Immunoresearch Fc Goat IgG polyclonal 109-005-098
Abcam FOXG1 Rabbit IgG polyclonal ab18259
DSHB Hb9 Mnx1 Mouse IgG1 monoclonal (81.5C10) 81.5C10-c
Sigma MAP2 Mouse IgG1 monoclonal (HM-2) M4403
R&D Systems mEphA4 Goat IgG monoclonal (SDL.3010) AF641
Cell Signaling Nanog Rabbit IgG monoclonal (D73G4) 4903T
Cell Signaling Oct-4A Rabbit IgG monoclonal (C20A3) 2840T
DSHB PAX6 Mouse IgG1 monoclonal (PAX6) PAX6
Cell Signaling phospho-Akt (S473) Rabbit IgG monoclonal (D9E) 4060S
ThermoFisher phospho-MYL9 (Thr18, Ser19) Rabbit IgG polyclonal PA5-17727
Cell Signaling phospho-S6 ribosomal protein (S235/236) Rabbit IgG monoclonal (D57.2.2E) 48585
Cell Signaling phospho-S6 ribosomal protein (S235/236) Mouse IgG2b monoclonal (E2R10) 62016S
DSHB puromycin Mouse IgG2c monoclonal (PMY-2A4) PMY-2A4-s
Cell Signaling RhoA Rabbit IgG monoclonal (67B9) 2117
Cell Signaling S6 ribosomal protein Mouse IgG1 monoclonal (54D2) 2317S
R&D Systems SOX2 Goat IgG polyclonal AF2018
Dako Tau Rabbit IgG polyclonal A0024
Invitrogen Tra-1-60 Mouse IgM monoclonal (2102Ep) ma1-023
Cell Signaling Tuberin/TSC2 Rabbit IgG monoclonal (D93F12) 3635
Sigma tubulin, acetylated Mouse IgG2b monoclonal (6-11B-1) T6793-2ML

	Jackson Immunoresearch HRP Goat IgG monoclonal (5A6-1D10) 211-032-171
Validation	Actin: by peer review, >680 citations and confirmed in our lab via WB band at appropriate size.<br>beta-III tubulin: by peer review, >360 citations and confirmed in our lab with specific neuronal labeling.<br>EphA2: by manufacturer, via EphA2 expressing and non-expressing cell lines, respectively and our lab by specific cell labeling.<br>Anti-Fc: Verified in our lab via staining of Fc patterned protein carpets.<br>FoxG1: by manufacturer, via FoxG1 overexpression in embryonic cortex and verified in our lab with distinct nuclear labeling<br>Hb9: by depositor, Tanabe et al., Cell 1998 and verified in our lab with specific nuclear labeling in neurons.<br>MAP2: by manufacturer immunofluorescent labeling specific to dendritic processes and confirmed by our lab.<br>EphA4: by manufacturer, via specific staining of embryonic rat rib cartilage primordium and our lab by specific cell labeling.<br>Nanog: by manufacturer, via specific immunofluorescent staining of pluripotent and differentiated cells, and confirmed by our by specific cell lab.<br>Oct4a: by manufacturer, via immunofluorescent staining of pluripotent and differentiated cells and verified by our lab with distinct nuclear labeling.<br>Pax6: by depositor, Kawakami et al., Mech Dev 1997 and verified by our lab with distinct nuclear labeling.<br>p-Akt: by manufacturer, via PI3K inhibition and insulin treatments, respectively and similar cell treatment by our lab.<br>p-MYL9: by manufacturer, via co-labeling w/ actin in HeLa cells and cell treatment validation by our lab.<br>p-S6: by manufacturer, via rapamycin and serum treatment, respectively and similar treatment by our lab.<br>p-S6: by manufacturer, via rapamycin and serum treatment, respectively and cell treatment by our lab.<br>Puromycin: Verified in our lab via protein synthesis inhibition and agonist experiments.<br>RhoA: by manufacturer, via cell line overexpression and WB assay and confirmed by our lab.<br>S6: by manufacturer, via WB band at appropriate size, as confirmed by our lab.<br>Sox2: by manufacturer, via specific staining in multiple neural-differentiated stem cell lines, and confirmed in our lab.<br>Tau: by peer review, >160 citations and specific neuronal staining by our lab.<br>Tra-1-60: by manufacturer, via immunofluorescent staining of pluripotent and differentiated cells and specific cell labeling by our lab.<br>TSC2: Verified in our lab via staining patterns in WT and no staining of TSC2(-/-) cell lines<br>Acetylated-tubulin: by peer review, >700 citations and specific neuronal staining by our lab.<br>HRP: by peer review, >200 citations and protein blot by our lab.

Eukaryotic cell lines

Policy information about cell lines

Cell line source(s)	All clones for patient-derived lines were generated via reprogramming from independent samples of patient skin fibroblasts. CRISPR-Cas9 genomic editing from stem cells was performed to generate multiple clones of isogenic control and TSC2(-/-) cell lines. WA09 (H9) and IMR90-4 lines were obtained via WiCell, Madison WI. CRISPR-Cas9 genomic editing from stem cells was performed on the IMR90-4 line in order to generate an additional isogenic heterozygous TSC2(+/-) line.
Authentication	All new cell lines were validated upon generation via karyotyping and Sanger sequencing at the mutation region in addition to each of the top twenty off-target sites as determined via crispr.mit.edu.
Mycoplasma contamination	All cell lines tested negative for mycoplasma upon generation and all culture work was performed in an antibiotic-free environment.
Commonly misidentified lines<br>(See ICLAC register)	No commonly misidentified cell lines were utilized in this study.

Human research participants

Policy information about studies involving human research participants

Population characteristics	This study involved the recruitment of a single male participant.
Recruitment	The patient was selected as he was severely affected by Tuberous Sclerosis Complex (TSC) and had a history of complex partial seizures, autism and expressive language delay, intellectual disability, as well as a large tuber burden. This patient was also selected because he had a heterozygous C>G point mutation at bp 972, which results in a premature stop at codon. This site was ideally positioned for further gene editing using Crispr.
Ethics oversight	Primary dermal fibroblasts were isolated from tissue acquired with approval from the University of Wisconsin-Madison Human Subjects IRB (protocol #2016–0979).

Note that full information on the approval of the study protocol must also be provided in the manuscript.
